# Priority use cases for antibody-detecting assays of recent malaria exposure as tools to achieve and sustain malaria elimination

**DOI:** 10.12688/gatesopenres.12897.1

**Published:** 2019-02-12

**Authors:** Bryan Greenhouse, Jennifer Daily, Caterina Guinovart, Bronner Goncalves, James Beeson, David Bell, Michelle A. Chang, Justin M. Cohen, Xavier Ding, Gonzalo Domingo, Thomas P. Eisele, Patrick J. Lammie, Alfredo Mayor, Nicolas Merienne, Wuelto Monteiro, John Painter, Isabel Rodriguez, Michael White, Chris Drakeley, Ivo Mueller, The Malaria Serology Convening

**Affiliations:** 1 Department of Medicine,, University of California San Francisco, San Francisco, CA, USA; 2 Consultant to UNITAID, Denver, CO, USA; 3 ISGlobal, Hospital Clínic - Universitat de Barcelona, Barcelona, Spain; 4 PATH, Seattle, WA, USA; 5 London School of Tropical Medicine & Hygiene, London, UK; 6 The Burnet Institute, Melbourne, Australia; 7 Intellectual Ventures, Bellevue, WA, USA; 8 Centers of Disease Control and Prevention, Atlanta, GA, USA; 9 Clinton Health Access Initiative (CHAI), Boston, MA, USA; 10 FIND, Geneva, Switzerland; 11 Center for Applied Malaria Research and Evaluation, Tulane School of Public Health and Tropical Medicine, New Orleans, LA, USA; 12 Institut Pasteur, Paris, France; 13 Tropical Medicine Foundation Dr. Heitor Viera Dourado, Manaus, Amazonas, Brazil; 14 Walter and Eliza Hall Institute of Medical Research, Parkville, Australia

**Keywords:** Malaria, Serology, Use Cases, Elimination

## Abstract

Measurement of malaria specific antibody responses represents a practical and informative method for malaria control programs to assess recent exposure to infection. Technical advances in recombinant antigen production, serological screening platforms, and analytical methods have enabled the identification of several target antigens for laboratory based and point-of-contact tests. Questions remain as to how these serological assays can best be integrated into malaria surveillance activities to inform programmatic decision-making. This report synthesizes discussions from a convening at Institut Pasteur in Paris in June 2017 aimed at defining practical and informative use cases for serology applications and highlights five programmatic uses for serological assays including: documenting the absence of transmission; stratification of transmission; measuring the effect of interventions; informing a decentralized immediate response; **and testing and treating *P. vivax *hypnozoite carriers.

## Background

### Challenges in eliminating malaria

As an increasing number of countries have intensified control efforts and are moving towards elimination of malaria, national malaria programs face new challenges understanding the nature and extent of residual transmission and tailoring their responses appropriately. As endemicity declines, transmission has been reduced and malaria has become more heterogeneous and is often concentrated in specific localities or populations ^1– 3^. Part of the response to this challenge, outlined in the WHO’s Global Technical Strategy ^4^, is strengthening malaria surveillance as a core intervention to better monitor and evaluate interventions. However, progress in reducing malaria burden globally is stalling in the face of contracting financial resources ^5^, and the need to maximize the efficiency of resource targeting and monitor impact is therefore increasingly critical. Accurate measurement of transmission can help programs to select and deploy optimal sets of interventions to appropriate areas more efficiently and subsequently monitor their impact ^6^. Among the more critical questions facing programs are: i) How to know when local transmission has been interrupted? ii) How to measure spatial variation in transmission risk accurately and efficiently in order to optimally deploy interventions? iii) How to efficiently monitor change in malaria transmission over time, whether due to deployed interventions or other factors? and iv) What additional strategies to deploy against *P. vivax*, in particular against the dormant liver stage (hypnozoite) that is undetectable with current diagnostics, but causes recurrent infection and perpetuates transmission?

Several tools and metrics, both parasitological and entomological, exist for measuring malaria transmission (reviewed previously ^7, 8^). In most countries, clinical case reporting systems are the backbone of malaria surveillance, and when implemented well can provide a good overview of where malaria is observed throughout the country. While an essential component of any malaria program, measurement of clinical cases alone has limitations as a surveillance strategy. First, cases are typically reported from health facilities, but transmission may be occurring elsewhere. Second, clinical case reporting will not include asymptomatic infections, (e.g. 9– 11), which may be driving transmission in certain contexts. Third, important sub-populations that may be contributing meaningfully to transmission may not come into contact with the formal health system, such as migrant laborers and those obtaining care in the private sector. Fourth, in many countries, data systems remain of poor quality and reporting may be incomplete.

As a result of these limitations, routine case reporting is often supplemented by malaria prevalence surveys. However, when transmission levels decline, obtaining prevalence data at a useful scale requires increasingly and prohibitively larger sample sizes ^12^. Other transmission metrics include the entomological inoculation rate and vectorial capacity; these are seldom measured beyond sentinel sites due to the complexity of implementation, limiting their usefulness for understanding patterns of transmission across a region. Novel solutions are thus needed to supplement existing tools for measuring transmission patterns across low transmission areas ^7^. Availability of flexible tools that can efficiently provide additional information regarding spatial and temporal variation in transmission would complement existing approaches and activities. Given the timelines for elimination that have been established in many countries, the development of such tools has become increasingly critical ^7, 13^.

### Potential advantages of measuring exposure

Measuring recent exposure to malaria is useful for understanding transmission risk in low transmission and elimination settings. The concept of exposure is different from incidence or prevalence in that it measures infection in the recent past (e.g. 6, 12, or 24 months) and not only new or ongoing infections. In other words, instead of identifying only individuals who are infected with *Plasmodium* blood-stage infections on the day of testing, an approach using exposure identifies individuals who have had at least one infection during a defined period in the recent past. Measuring exposure has advantages over other methods for low transmission settings. In these settings, when using diagnostic methods based on detection of circulating parasites, even the most sensitive molecular assays will only detect the minority of actively infected individuals because many infections will have hard-to-detect low parasite densities, or in the case of *P. vivax* liver-stage-only infections, which are simply undetectable. Moreover, even the most sensitive tests will miss individuals who have recently cleared infections but may have contributed to and thus reflect transmission.

Population surveys over large geographic areas (e.g., an entire eliminating country or province) can only test a small fraction of the total population over a narrow window of time. This limitation means that determination of geographical areas with higher transmission compared to other regions will require large sample sizes (often exceeding 5,000-10,000) if parasite prevalence is low and only active infections are evaluated. Sample size requirements are potentially even larger if high spatial resolution (e.g., village or sub-district level) is required for efficient targeting of interventions ^8^, and similar limitations exist for evaluating demographic risk groups. In comparison, a sensitive test for recent exposure, by capturing infections that occur over a period of time rather than at a single time-point, will require a smaller sample size to yield the same information on whether transmission has been occurring, and if so to what degree. Alternatively, instead of decreasing the size and cost of a survey, by measuring recent exposure a survey with a fixed budget and sample size can yield greater spatial resolution and thus more information about how transmission varies across the region of interest than would be possible with conventional diagnostics. 

Another highly pragmatic benefit of measuring exposure instead of active infection relates to the timing of surveys. Optimally, parasite prevalence surveys are conducted during the malaria transmission season, coinciding with heavy workloads for programs and challenging conditions during the rains. The timing of malaria exposure surveys is more flexible because the test captures information over a span of time that is longer than the time of the survey, allowing programs to conduct a survey after the rainy season, for example.

These efficiencies have the potential to yield more information about transmission patterns without requiring more financial and operational investment than is already being made by elimination programs. Programs have limited financial resources and face significant operational constraints (e.g. insufficient human resource capacity, transport, and logistics). New diagnostic methods that allow sensitive detection of recent exposure and that can be easily implemented in low-resource settings could play a major role in measuring progress toward malaria elimination in these settings and in aiding the design and development of interventions.

### Antibodies are the best operationally-relevant tool for measuring exposure

The total *Plasmodium* proteome is thought to contain >5,000 distinct proteins, many hundreds of which have been shown to be recognized by the human immune system. Antibodies to many of these proteins are efficiently induced and boosted by *Plasmodium* infections and persist in decreasing titers over several months to years after the clearance of the blood-stage infections ^14– 16^, making them potential markers of not only current but also recent and historic exposure.

In low-transmission settings or in populations with limited prior exposure (e.g. children), antibodies have been shown to be strongly linked to exposure ^17^. Antibody responses have also been shown to correlate closely with transmission intensity and for historically low- and moderate-transmission settings are a potentially more efficient transmission measure than infection prevalence or incidence rate ^18, 19^. Antibodies can be measured with relatively simple assays, including inexpensive laboratory-based assays and point-of-contact tests similar to those developed for other infectious diseases such as HIV, influenza and leishmaniasis ^20– 22^. Antibody detection may thus be more sensitive, and potentially lower-cost than nucleic acid detection for identifying the ongoing presence of malaria transmission in populations. 

The antibody response to each parasite protein has different kinetics, i.e. different rates of acquisition, boosting, and decay, and researchers have now started to study these antibody kinetic profiles for many antibodies to *P. falciparum* and *P. vivax*, and to characterize their suitability for estimating recent exposure in different age groups and transmission settings ^23^. Several ongoing efforts are using this information to identify antibody signatures that are reliable, quantitative measures of recent exposure to malaria parasites ^24– 28^. In parallel with biomarker identification, antigen production methods, analytics and technology platforms have advanced sufficiently to generate malaria antibody assays that will be field deployable in the near future. Detailed consideration of assay design and development is beyond the scope of this article, but has been recently reviewed ^29^. An important advance, is the measurement of multiple antibody responses in combination to increase the accuracy of estimates of recent exposure for each individual screened. The ability to obtain more accurate data on recent exposure from each individual by using antibody signatures allows for more accurate estimates for populations and for more flexibility in sampling, providing a departure from more cumbersome age stratified serological approaches used in the past.

### Malaria serology convening, Paris, 6–7 June 2017

In light of global malaria elimination targets and recent progress in malaria serology research, a meeting was convened in June 2017 to understand the state-of-the-art in development of antibody assays for malaria surveillance (e.g., biomarker identification, statistical analysis, diagnostic technology platforms, and operational work). A total of 39 representatives from 28 institutions participated. Given the scientific and operational advances, it was timely to assess the range of applications with a view to prioritizing them for further investment and development. As such, a key aim of the meeting was to review potential use-case scenarios, including experiences using and evaluating serological biomarkers in control and elimination programs. This paper synthesizes and builds upon the discussions of use-case scenarios with an aim towards focusing research and investment on those applications that will have the greatest impact on malaria transmission reduction and elimination. The use cases described herein are purposely technology platform-neutral, do not refer to specific detection targets, and, with one exception, are relevant to both *P. falciparum* and *P. vivax*. It should also be noted that whilst all use cases refer to measurement of recent exposure using antibodies, the period of time over which past exposure will be informative may vary by use case and epidemiologic setting as well as the biological and technical constraints of the assay. As such, in depth consideration of the specific period of time for recency is beyond the scope of this review and not specified below, but in general was agreed to be in the range of six months to three years.

## Use cases for serological markers of exposure

### Spectrum of applications

Many uses of malaria antibody assays have been described previously (reviewed in 30– 32). These vary greatly in their purpose; while some are purely surveillance tools for characterizing transmission, others are used to trigger a control approach or measure the effect of an intervention. These uses also differ in how they are applied – at the individual or population level – as well as where on the transmission spectrum they are most relevant. Recently, additional approaches have been proposed, including the use of malaria antibody assays to target interventions to specific populations, and at least for *P. vivax,* to individuals.

To date, the various potential use-case scenarios have neither been accurately described nor prioritized. A detailed understanding of which functions the test is expected to perform and on what target population is a pre-requisite for the development of detailed target product profiles, and consequently for further investment and actual product development. In the diagnostics industry, these decisions are typically made based on market research and profitability analysis, but for a global health market of limited size, investment decisions in new product development should be informed by the health impact associated with specific use cases in addition to market considerations. If a sufficiently robust case can be made, the funding mechanisms that drive malaria diagnostics markets (predominantly bilateral and multilateral aid agencies) provide a conducive environment for providing needed support.

**Figure 1.**
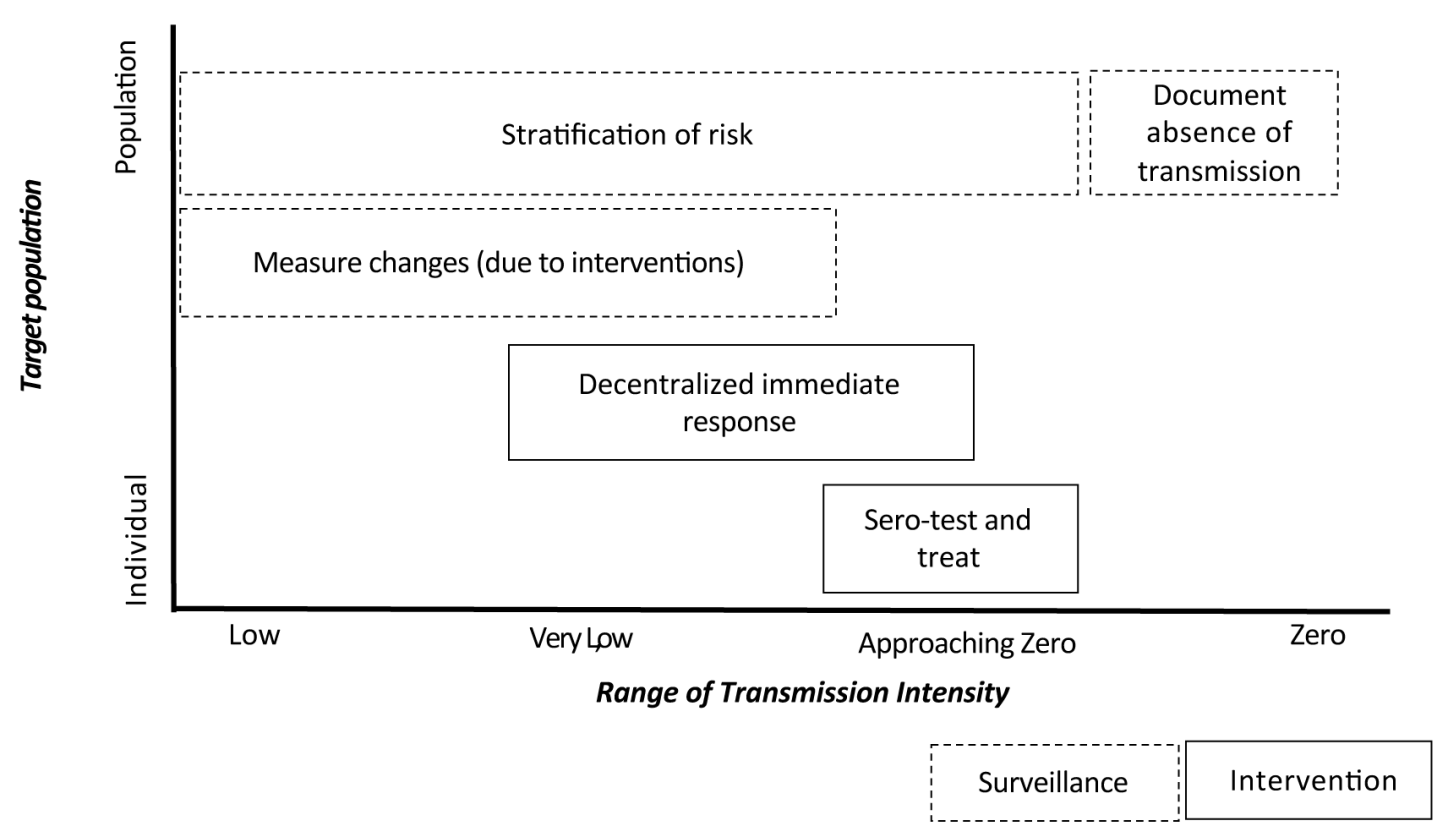
Priority applications of serological markers of exposure.

As such, we consider the use-case scenarios for malaria antibody assays through two lenses. Firstly, does testing for exposure offer compelling advantages, or additional value, over more routinely used metrics? Secondly, does the application provide critical information to control and elimination programs? Using these filters, the following five use-case scenarios are reviewed: i) documenting the absence of transmission over a given geographic space; ii) stratification of transmission level; iii) measuring the impact of interventions and monitoring changes in malaria transmission; iv) decentralized immediate response; and v) serological testing and treatment for *P. vivax* hypnozoites ( Figure 1). 

Whilst discussions focused primarily on low transmission, pre- and peri-eliminations settings, whether exposure testing in general and some of these same use cases in particular may also have utility in higher transmission settings (e.g. measuring changes in transmission that follow interventions but are not detectable by parasite prevalence measurements ^8^, understanding population immunity to help identify populations at high risk ^32^) was also discussed.

### Priority use-case scenarios of serological markers of exposure

***Use Case 1: Document absence of transmission***. One end of the transmission spectrum is the absence of transmission in a defined area ( Table 1). Malaria antibody tests with appropriate performance characteristics would provide a sensitive tool for quickly confirming the absence of transmission via the absence of antibodies specific for recent malaria exposure ^30, 32^. For example, a program may have no passively detected indigenous cases in an area and want evidence from another reliable approach about the absence of transmission before scaling back an intervention (e.g. IRS) or shifting focus to prevention of reintroduction. In this use-case scenario, antibody testing would be used to document the absence of transmission over a defined period of time, at the subnational or district level, along with routine passive case detection and case investigation (which are required for WHO certification of elimination) and metrics like annual blood slide examination rates. As with all of the other population-based use cases described below (Use Cases 2-4), screening may occur through surveillance of the general population or within relevant sentinel populations chosen because they are at highest risk or are more efficient to screen (e.g. migrants/mobile populations, pregnant women, school children). Depending on the population screened, antibody-detection testing may occur in participants captured by active screening (e.g., cross-sectional surveys) or passively through sentinel populations (e.g., screening at health facilities or prenatal clinics). This use case does not necessarily require a point-of-contact test, since these programmatic surveys can be evaluated by a central laboratory. A point-of-contact format may also be considered, in particular if confirmation of ongoing transmission is linked to an immediate, decentralized programmatic response (see Use Case 4 below). Use Case 1 will require an antibody marker with high specificity for recent exposure, and, depending on the elimination related goals, species specificity ^33^. Some antibodies can persist for extended periods in some individuals ^34^, which may lead to false positives. Therefore, careful selection of antibodies with relatively short half-lives but consistent boosting in those exposed will be required. Combining antigens with different kinetic profiles in the same test may be possible (either via multiplex assays or separate targets on point-of-contact tests) which could improve the accuracy of time-since-exposure estimates. Similarly, targeting of testing to known high-risk groups may further the effectiveness of this approach.

**Table 1.** Priority use-case scenarios for serological markers of exposure.

Use-case scenario	Document absence of transmission	Stratification of transmission	Measure impact of interventions	Decentralized immediate response	*P. vivax* sero test and treat
Problem addressed	Demonstrate the absence of local transmission; always in complement to other surveillance (e.g. passive case detection)	`Identifys where is transmission happening and at what level	Detemines the impact of an intervention, either new or established?	Informs an immediate response by teams in the field.	Identifies individuals with *P. vivax* infection during a specified time period, including silent carriers (hypnozoites, asymptomatic blood stage) for radical cure.
Action taken based on result	No evidence of transmission triggers shift to prevention of introduction; evidence of transmission would trigger a response	Mapping of risk exposure in order to guide intervention strategy	Policy recommendation; continue / adjust intervention. Reporting on impact (internal and external).	Varies, depending on context, e.g., IRS, MDA, education	G6PD testing and treatment with primaquine/tafenoquine.
Individual or population level	Population	Population	Population	Population (smaller)	Individual
Operational unit of implementation	National, subnational and district	National, subnational, district.	Subnational, district	District, village, defined demographic/risk populations	Village, district, defined demographic / risk populations
Transmission level	Approaching zero or zero	Medium (with heterogeneity) to very low	Low to approaching zero	Low to approaching zero	Low to approaching zero
Requires point of contact test	No	No	No	Yes	Yes
Alternatives	Routine case reporting, infection prevalence surveys (RDT, microscopy, molecular), and metrics such as positivity rates and annual blood examination rate	Routine case reporting; infection prevalence surveys (RDT, microscopy, molecular)	Routine case reporting; infection prevalence surveys (RDT, microscopy, molecular)	Active case detection (RDTs, Highly Sensitive RDTs, Microscopy, Molecular)	No alternative for identifying hypnozoites; MDA as an alternative intervention

G6PD, glucose-6-phosphate dehydrogenase; IRS, indoor residual spraying; MDA, mass drug administration; RDT, rapid diagnostic test.

***Use Case 2: Stratification of transmission***. As malaria endemicity drops to low or very low levels, transmission becomes increasingly heterogeneous, with pockets of residual transmission surrounded by large areas with little or no local transmission ^1^. In these settings, blanket coverage of interventions may not be cost-effective, and surveillance for stratification becomes an integral part of control and elimination programs ^4, 35^. In order to target interventions effectively, national programs need to stratify the country and populations into different transmission levels and to use this information to guide interventions.

In areas where transmission is highly focalized and routine surveillance coverage potentially incomplete, antibody data can augment clinical case data to provide a more granular picture of recent transmission (e.g. to direct locally targeted interventions). This may be especially true if the incidence of asymptomatic infections is high, as these infections would be missed by case reporting systems. In this use-case scenario, seroprevalence data could be added to geo-spatial mapping programs (e.g. the Malaria Atlas Project), facilitating use of these data for programmatic stratification and action. For this use case a lab-based or point-of-contact test are both possible. Centralized testing may appeal because stratification usually occurs over higher administrative levels and would likely coincide with annual program activities. Stratification will require antibody markers with optimal balance between sensitivity and specificity for recent exposure across a range of transmission levels (e.g. from no to moderate transmission), as well as corresponding guidance on sampling. This will enable targeting of resource intensive interventions to areas of highest transmission or areas highly susceptible to interruption of transmission, while enabling a scale-back or redirection in other areas.

***Use Case 3: Measure impact of interventions***. Ideally, programs would have the capacity to routinely measure the impact of interventions to ensure their continued effectiveness. However, existing surveillance systems often do not provide sufficient information on effectiveness despite the millions of dollars spent on malaria control interventions every year.

For impact evaluation purposes, antibody markers of exposure can be an effective measure. In practice, antibody testing would likely be used in two settings: 1) when new interventions are being piloted or implemented for the first time; and 2) intermittently thereafter to monitor the real-world effectiveness of these measures, especially when there is reason to suspect potential compromise of effectiveness.

The Garki project is a classic example of the utility of using antibodies to evaluate changes in transmission intensity after initiation of interventions ^36^. More recently, studies are beginning to use antibodies more specific for recent exposure, i.e. that have shorter serum half-lives. For example, operational research on focal mass drug administration (MDA) and vector control in Namibia is using antibody data that capture recent exposure as a study outcome ^37^. Serological test characteristics for this use case are similar to those required for the risk stratification use case above.

***Use Case 4: Decentralized immediate response***. In this use case, malaria teams would use point of contact antibody tests to survey a small proportion of the population, and results would inform an immediate action at the focal level (village, household, defined high risk populations, etc.). As serological positivity is expected to exceed infection prevalence, antibody testing would generate more granular data on which to base decisions. Operationally, the response to positive antibody results could vary (e.g. focal larval control, IRS, focal testing and treatment or focal MDA, education, etc.) and would be triggered by a predefined threshold of positivity in the sampled population. The requirement for decentralized decision-making and real-time data to inform immediate response distinguishes the current use case from those discussed earlier in that it requires a point-of-contact test.

While such interventions could be conducted with parasite antigen-detecting rapid diagnostic tests (RDTs), especially highly sensitive RDTs, a point-of-contact antibody detection tests could have a higher sensitivity in detecting recent events and thus potentially require a smaller sample size and/or be used on different target populations, i.e. known high-risk groups such as forest workers in certain South East Asian settings who may harbor hypnozoites. Similar types of interventions have also been attempted with molecular methods ^38, 39^; however, these are typically cumbersome and costly to implement in the field, and turnaround time, even with mobile laboratory set-ups, is not as fast as with point-of-contact tests. In the case of *P. vivax,* serological markers may be able to identify hypnozoite carriers (see Use Case 5). While we are not aware of this type of intervention being currently deployed in malaria using antibody tests, similar approaches have been tried with RDTs ^40^ and molecular methods ^39^ with mixed results, and are routinely implemented with antibody testing in other neglected tropical diseases (for example the transmission assessment surveys for filariasis ^41^).

***Use Case 5: P. vivax sero-test-and-treat***. Currently, there are no tests to assess whether an individual is infected with hypnozoites, the dormant, liver-stage forms of *P. vivax* (and *P. ovale*) that cause relapses and are asymptomatic ^42^. Hypnozoites, which can re-activate months or even 1-3 years after clearance of the primary blood-stage infection ^43^, account for up to 80% of all *P. vivax* infections and are thought to contribute substantially to transmission ^44^. Hypnozoites require radical cure with primaquine or tafenoquine to avoid recurrent infections, which potentially result in symptomatic disease and transmission to mosquitoes. By definition, dormant stages are not detected by blood-stage infection tests, thus, with current tools programs are faced with a choice between missing carriers with mass test and treatment (MTAT) or treating whole populations with MDA ^45^. The ability to safely perform MDA is limited in part due to the risk of 8-aminoquinoline-induced haemolysis in glucose-6-phosphate dehydrogenase (G6PD)-deficient people ^46^. This is of particular concern in low endemicity settings where up to 85–95% of people treated do not require treatment, as they do not have hypnozoites, but for safety reasons need to be tested for G6PD deficiency prior to receiving 8-aminoquinolines and monitored for compliance, a challenging and resource-intensive undertaking. An alternative parasite clearance intervention is MTAT, but without a sensitive assay that identifies people carrying only hypnozoites this approach is likely to be ineffective ^47^ because it would miss a large proportion of the reservoir ^48^. As all *P. vivax* strains—except for the hibernans strains currently thought to be restricted to the Korean peninsula—cause primary blood-stage infections followed by a primary relapse within 1 – 9 months ^43^, serological markers able to detect a *P. vivax* (blood-stage) infection in around the previous 9 months could act as an effective proxy for potential hypnozoite carriage. 

In this scenario, a population is screened and those individuals testing positive for recent *P. vivax* exposure with a point-of-contact test would receive radical cure (unless contraindicated or recent treatment can be confirmed). This strategy is denoted serological testing and treatment (SeroTAT). While not all antibody-positive individuals will harbor hypnozoites, serological screening will avoid most of the large-scale overtreatment associated with MDA, and therefore help programs implement parasite treatment interventions that lead to clearance of hypnozoites. Programmatic implementation is likely to require a test that can detect recent exposure to *P. vivax* with high sensitivity (to assure efficacy) and good specificity to avoid over-treatment. While this intervention has yet to be fully evaluated, transmission decline and eventual elimination of malaria in some Southern Brazil localities in the 1980s was attributed at least in part to treatment guided by malaria serology ^49^. Serological testing was also used to target focal MDA programmes for the elimination of *P. vivax* in Jiangsu province, China from 2000–2009 ^45^. Serological test and treat approaches may also be an appropriate intervention to eliminate residual pockets of *P. ovale* transmission and could be useful for the screening of returning travelers or migrants that move from a *P. vivax* endemic area to a no longer endemic (but receptive) area. 

### Toward implementation of antibody-detecting surveillance and response

Currently, malaria antibody tests have primarily been used in the research context, and a number of steps are required if malaria programs are to integrate antibody testing more broadly into surveillance and response. Among these steps are proof-of-concept studies on the impact of interventions involving the use of malaria antibody tests (e.g. decentralized immediate response, SeroTAT), and development of operational strategies for transmission monitoring use cases (e.g. sampling strategies). Simultaneously, researchers continue to refine biomarkers and analytical methods, while developers have begun work to migrate these assays to appropriate technology platforms. Importantly, quality assurance (including the establishment of appropriate standards) should be considered comprehensively at this stage in order to ensure that results are reliable and comparable ^50^. 

In addition to malaria antibody tests that provide sufficiently reliable answers to the key questions faced by programs, it is important that any new tools address the context and conditions of use. Elimination programs have limited finances and face significant operational constraints, such as the scarcity of trained staff and transportation bottlenecks, especially at subnational levels. With the exception of *P. vivax* SeroTAT (Use Case 5), there are existing transmission metrics that might perform the same role as malaria antibody testing, and it is important that any new test is considered in this context. When used in surveillance, monitoring, or stratification (Use Cases 1–3), antibody testing will most likely be used in conjunction with other tools, and therefore programs must critically consider the value added in order to justify the investment of limited time and resources. Additional investment required will vary considerably depending on how tests are integrated into surveillance activities. In some cases, the addition of these tools will add minimal cost and effort, e.g. antibody testing of dried blood spots already being collected as part of a cross-sectional survey or in the context of already existing sampling schemes (e.g. antenatal clinic visits). In other cases, such as point-of-contact testing for decentralized immediate response, the unit cost per test may be higher; however, these may be offset by other savings and operational advantages. The potential utility and applications of antibody testing are likely to vary by regions and populations, influenced by a range of factors such as transmission epidemiology, local resources and infrastructure, population size and mobility, and access. In some settings serology will be a valuable addition, in others it may be less useful.

Across all applications, expected situations of use will include resource poor settings, where environmental conditions for both specimen storage and performing assays may not be well controlled. The tests or sample collection systems must therefore be robust enough to withstand extreme conditions. Because sample collection, and in many cases testing itself, is likely to be performed by field workers with little training, ease-of-use is paramount. These requirements will likely be easier to achieve for Use Cases 1–3, which do not require a point-of-contact testing (e.g. using dried blood spot samples) than for Use Cases 4 and 5, which require a field stable point-of-contact test. The use of a point-of-contact test and remote data collection may be an advantage in remote locations, or in areas among populations with difficult or infrequent access (e.g. geographically remote settings, conflict and disaster settings, mobile populations), but is only essential if positive test results are followed by an immediate programmatic response (either individual treatment or implementation of population-based interventions, i.e. Use Cases 4 and 5). The test result must also be unambiguous, providing actionable data to the relevant end-user, be it a field worker reading a binary a point-of-contact test, or a district manager receiving a report from the laboratory. While the turn-around-time for antibody tests triggering immediate interventions must be as rapid as possible, an important consideration for other use cases may be simplifying batch processing for laboratory-based methods to make these tests more accessible to programs with limited resources while still providing data within the appropriate timeframe needed for decision-making. 

Finally, it will be important to engage global and national malaria programs, affected communities, non-governmental organisations, donor agencies and malaria research institutes on the concept of measuring exposure and various use case scenarios for malaria antibody tests. Their feedback is important to further prioritization and target product profile development; however, since antibody-detection testing is a departure from the traditional focus on direct parasite detection, a thoughtful and proactive engagement strategy is needed. Modeling of likely cost-benefits of resource targeting and acceleration of elimination will be an important aspect of this, along with building a track record and evidence base through demonstration studies.

As discussed, the driving rationale for antibody testing is its pragmatic, practical advantages, for example facilitating more nimble surveillance by reducing sample sizes required for precise, actionable data; the ability to have a rapidly deployable test that provides timely intelligence in the field to target local interventions; and its unique potential to identify hypnozoite carriers. Given the contextual challenges that malaria programs face, and the range of malaria typologies that exist today, antibody testing is likely to have a meaningful impact on malaria elimination efforts on multiple fronts. Current technological advances suggest the potential for development of operational, standardized antibody-detecting tools for malaria surveillance and response in the short term. Validation of at least one such approach would likely pave the way for a set of antibody-detection assays to address the five use cases outlined here. 

## Data availability

No data are associated with this article
