# Supplementary material for: Priority use cases for antibody-detecting assays of recent malaria exposure as tools to achieve and sustain malaria elimination
Source: Gates Open Res. 2019 Feb 12;3:131. [Version 1] doi: 10.12688/gatesopenres.12897.1 (PMC6545519; doi:10.12688/gatesopenres.12897.1)
Supplement: Peer review history

# Peer review: Reviewer response for version 1

**DOI:** 10.21956/gatesopenres.13993.r27127

**Published:** 2019-07-11

**Authors:** Ronald Perraut

**Affiliations:** 1 Unité d’Immunologie, Institut Pasteur de Dakar, Dakar, Senegal

The Open Letter “Priority use cases for antibody-detecting assays of recent malaria exposure as tools to achieve and sustain malaria elimination” is a team global report with a synthesis of results and discussion of a meeting in Institut Pasteur. This paper emphasizes the central role of Ab responses to several Ag targets as a very central potential capacity for malaria survey. Ab signature (short- or long-lived) remains the most sensible way to detect parasite circulation. It is presently more sensible and much easier to use than molecular diagnosis.

- The 5 scenarios discussed are well described and clearly summarized.
- The scenario Case 2 for stratification of transmission is a great challenge as detectable submicroscopic and/or asymptomatic infections are highly variable (time and location). Mapping such risk would require sustained sentinel site follow-up with relevant geographic representativeness for a given region. This is illustrated by scenario Case 5 with *P. vivax* appearing in unexpected areas travelling with hypnozoite carriers. MDAT elimination procedure is a risky strategy and ethically difficult to improve.
- As a last point, I entirely agree with the Case 3 summary: measure of impact of intervention should be highly implemented. Action without evaluation of the action is a dead-end procedure. There is a line for monitoring & evaluation that is supposed to be 5% of the Global Fund budget in the recipient countries. A real use of this part of funding would really help.

I confirm that I have read this submission and believe that I have an appropriate level of expertise to confirm that it is of an acceptable scientific standard.

# Peer review: Reviewer response for version 1

**DOI:** 10.21956/gatesopenres.13993.r27198

**Published:** 2019-06-07

**Authors:** Kingsley Badu, Christian Owusu, Thomas Addison

**Affiliations:** 1 Department of Theoretical and Applied Biology, Kwame Nkrumah University of Science and Technology (KNUST), Kumasi, Ghana; 2 Kumasi Center for Collaborative Research for Tropical Medicine, Kumasi, Ghana; 3 Theoretical and Applied Biology, Kwame Nkrumah University of Science and Technology (KNUST), Kumasi, Ghana

The paper “Priority use cases for antibody-detecting assays of recent malaria exposure as tools to achieve and sustain malaria elimination” presents and discusses five scenarios where the use of serological markers of malaria exposure provides clear advantages over currently-utilised tools. The idea of measuring recent exposure to infection is one that has been rapidly gaining traction, in the last 20 years, especially with the success of malaria control efforts and the accompanying drop in transmission intensity in many places.

 I have been involved in measuring malaria transmission intensity for well over 15 years; I have first hand experience of how difficult it is to use the conventional tools such as the entomological inoculation rates when transmission is low. Thus, I very much appreciate the role of serology as a proxy for malaria transmission. A major issue has to do with the choice of which antibodies to use for what purpose; because over 5000 proteins have got their own kinetics and must be chosen very carefully. However with the current advancement in multiplex antibody detection platforms, sufficient progress has been made towards elucidating antibody kinetic profiles for the suitability of measuring recent exposure. All these have been clearly discussed in this paper.

 What perhaps is still a challenge is standardizing methodology that will make it possible for using antibody based surveillance system application in national malaria control programs. On this too the authors give us hope that these are being developed. While the paper does not delve much into the technical aspects surrounding the implementation of an antibody-based surveillance system, the five scenarios discussed adequately cover the most likely situations in which such a system would prove most useful.

 It is also gratifying to note that the authors acknowledge the existence of transmission metrics that perform the same role as the proposed malaria antibody testing system and stress importance of taking this into account when trying to justify the implementation of any new test. Given the scarcity of resources in the malaria-endemic world, this is a critically important point that should always be taken into account when initiating a new intervention. Because in areas of Africa where malaria transmission intensity is still very high, some of the conventional tools are still very useful.

We confirm that we have read this submission and believe that we have an appropriate level of expertise to confirm that it is of an acceptable scientific standard.

# Peer review: Reviewer response for version 1

**DOI:** 10.21956/gatesopenres.13993.r27126

**Published:** 2019-05-28

**Authors:** Karen Kerkhof

**Affiliations:** 1 Department of Biomedical Sciences, Institute of Tropical Medicine, Antwerp, Belgium

There has been considerable progress in recent years towards developing tools for serosurveillance (including antigens and platforms) and understanding their utility and value in populations. This report entitled “Priority use case for antibody-detecting assays of recent malaria exposure as tools to achieve and sustain malaria elimination” covers this important topic of serosurveillance tools for targeting malaria with the focus on defining the practical and informative use cases for malaria serology.

 The focus of this paper is to define the five different use cases that can be applied in surveillance, monitoring or stratification (epidemiology, use cases 1-3) and individual treatment or implementation of population-based interventions (use cases 4 and 5) with the focus on both *P. falciparum* and *P. vivax*. Use cases 4 and 5, in particular, are of great importance and indicate the strength of this paper, since there has been less attention on mass drug administration (MDA) specifically for *P. vivax* or *P.ovale. *These species become an ultimate challenge for most eliminating countries. They are more resilient than *P. falciparum* because *P. vivax *is able to persist in a dormant liver (hypnozoite) stage and relapse over long intervals of months to years.

 Citing the importance, but also the knowledge gaps within the surveillance tool development, especially about how to overcome the gap between mass test and treatment (MTAT) and MDA, are clearly discussed. Although, as already mentioned by the previous reviewer, not much emphasis is placed on how to address the technical issues that may be encountered in the development process and perhaps have strengthened the paper, this is still a high-level paper. Furthermore, the authors use strong references and explain each interest and necessity of the five use cases very correctly and concisely. Mentioning the well-known Garki-project with the successful application of MDA as well as referring to other points on the Global Malaria Eradication program has not been overlooked.

I confirm that I have read this submission and believe that I have an appropriate level of expertise to confirm that it is of an acceptable scientific standard.

# Peer review: Reviewer response for version 1

**DOI:** 10.21956/gatesopenres.13993.r27130

**Published:** 2019-05-22

**Authors:** Eun-Taek Han

**Affiliations:** 1 Department of Medical Environmental Biology and Tropical Medicine, School of Medicine, Kangwon National University, Chuncheon, South Korea

In tropical and subtropical areas where malaria is endemic, prioritization of serological markers might be important together with asymptomatic case detection and relapse case control in cases of *Plasmodium vivax*. By the elimination or control of *P. falciparum* in some areas, even though some areas have increased numbers of *P. falciparum* cases, non- *falciparum* parasites including *P. vivax* in SE Asian countries and *P. ovale* in African countries have exponentially increased recently. For the control of those types of malaria, antibody-based detection systems or novel marker discovery are the most important in the pre-elimination stage. For that, this manuscript pointed out sequential control steps or practical implementation platform by 5 cases. By Dr. Ivo’s group, novel serological marker discovery has extensively progressed from blood- and liver-stage parasites. Thus we are promised to find novel serological markers for recent exposure or relapse within a few years. I think this manuscript has very important information on the strategy for the control or pre-elimination stage of malaria in endemic countries.

I confirm that I have read this submission and believe that I have an appropriate level of expertise to confirm that it is of an acceptable scientific standard.

# Peer review: Reviewer response for version 1

**DOI:** 10.21956/gatesopenres.13993.r26928

**Published:** 2019-02-15

**Authors:** G. Dennis Shanks

**Affiliations:** 1 School of Public Health, University of Queensland, Brisbane, Queensland, Australia; 2 Australian Defence Force Malaria and Infectious Disease Institute, Enoggera, Queensland, Australia

As one approaches malaria elimination, surveillance becomes increasingly important. The paper by Greenhouse *et al.* titled “Priority use cases for antibody-detecting assays of recent malaria exposure as tools to achieve and sustain malaria elimination” is a report of a scientific meeting in Paris in June 2017. The paper’s stated purpose is to define practical and informative use cases for malaria serology; it achieves this goal by defining and discussing five use cases across a range of epidemiology and programs. 

 The paper is necessarily generic and agnostic as to any particular platform or test. There are sufficient references to recent review articles to provide the reader with specific details they may require for their own situation. There is a reference to malERA with this paper being a further development of what surveillance tools are important for malaria elimination. Better than most meeting reports, it summarizes what must have been detailed discussions looking at a wide variety of options and epidemiology. The paper maps out what is needed and lacks the scope to discuss how to address the complex technical issues.

 Of the five use cases, I would especially note the fifth one: serological testing and treatment for *P. vivax*. The last parasites in any area will be relapsing species who have evolved to survive transmission gaps in seasons and populations. The challenge of killing the last latent hepatic parasite (hypnozoite) is great and will only increase in importance. The authors do a good job explaining this challenge and how the current gap between mass test and treat (MTAT) and mass drug administration (MDA) might be bridged with future serological testing.

I confirm that I have read this submission and believe that I have an appropriate level of expertise to confirm that it is of an acceptable scientific standard.
